# Differential gene expression and AKT targeting in triple negative breast cancer

**DOI:** 10.18632/oncotarget.27026

**Published:** 2019-07-09

**Authors:** Feng-Mao Lin, Susan E. Yost, Wei Wen, Paul H. Frankel, Daniel Schmolze, Pei-Guo Chu, Yate-Ching Yuan, Zheng Liu, John Yim, Zhen Chen, Yuan Yuan

**Affiliations:** ^1^ Department of Diabetes Complications and Metabolism, City of Hope National Medical Center and Beckman Research Institute, Duarte, CA, USA; ^2^ Department of Medical Oncology and Therapeutic Research, City of Hope National Medical Center and Beckman Research Institute, Duarte, CA, USA; ^3^ Department of Surgery, City of Hope National Medical Center and Beckman Research Institute, Duarte, CA, USA; ^4^ Department of Biostatistics, City of Hope National Medical Center and Beckman Research Institute, Duarte, CA, USA; ^5^ Department of Pathology, City of Hope National Medical Center and Beckman Research Institute, Duarte, CA, USA; ^6^ Bioinformatics Core Facility, City of Hope National Medical Center and Beckman Research Institute, Duarte, CA, USA

**Keywords:** AKT, differential expression, AKT targeting, TNBC

## Abstract

**Background:** Metastatic triple negative breast cancer (mTNBC) is a heterogeneous disease with poor prognosis. Molecular evolution of TNBC through chemotherapy selection pressure is well recognized but poorly understood. PI3K/AKT/mTOR is one of the most commonly identified oncogenic-driver pathways in breast cancer. The current study is designed to understand the genomic and transcriptomic changes, focusing on the PI3K/AKT/mTOR pathway alterations in paired primary and metastatic TNBCs.

**Results:** Genomic analysis of 7 paired specimens identified 67 known mutations including those from the following signaling pathways: cell cycle, p53, PI3K/AKT/mTOR, RAS/MAPK, and RTK/GF. Principle coordinate analysis (PCoA) identified 4 distinctive molecular groups based on the gene expression patterns of PI3K/AKT/mTOR pathway. Key differentially-expressed genes included AKT3, GSK3B, GNA11, PI3KR1, and GNAQ. Importantly, AKT-targeted therapy showed efficacy in a patient-derived xenograft (PDX) model of TNBC *in vivo*.

**Conclusion:** Genomic discordance of paired primary and metastatic TNBCs was identified, with significant increase in tumor proliferation pathways seen in metastases. Among the differentially expressed genes, AKT3 can potentially serve as a target for novel combination therapy for treatment of metastatic TNBC.

**Methods:** Paired specimens from 10 patients with TNBCs were identified through an IRB-approved protocol (2002–2015). FoundationOneTM sequencing was performed for genomic profiling, and Affymetrix Human Genechip 2.0st was used for mRNA expression profiling. The similarity among samples was calculated based on Pearson correlation coefficients, which were used to construct hierarchical clustering and heat maps.

## INTRODUCTION

Triple negative breast cancer (TNBC) accounts for 15–20% of all breast cancers and is characterized by poor overall survival upon disease relapse. Unlike hormone receptor positive or HER2/neu positive breast cancer (BC), there is no effective targeted therapy for treatment of TNBC with the exception of those with germline BRCA1/2 mutations [1–5] or programmed death ligand-1 (PD-L1)-expressing TNBCs [6–8]. Despite these recent advances which led to moderate improvement in progression-free survival, the overall survival of patients with mTNBC has not changed. Therefore, there is still an unmet need to identify effective therapy targeting oncologic drivers.

In order to develop effective targeted therapy, it is critical to identify targetable genomic or transcriptomic drivers accountable for chemotherapy resistance. However, this is highly challenging for TNBC because these tumors are very heterogeneous, with at least four molecular sub-types proposed to date based on transcriptomic mRNA expression [9–11]. The molecular classifiers of Lehmann/Pietenpol [9, 10] defines six molecular subtypes of TNBC: basal-like 1, BL1; basal-like 2, BL2; mesenchymal, M; mesenchymal-stem-like, MSL; immune-modulatory, IM and luminal androgen receptor, LAR. The Burstein classifier defines 4 molecular subtypes of TNBC: basal-like immune-activated, BLIA; basal-like immunosuppressed, BLIS; luminal androgen receptor, LAR; and mesenchymal, MES [11]. Although these sub-types have deepened our understanding of the complexity of TNBC tumor biology, the associated molecular classifiers have not yet been incorporated in any routine clinical practice, nor have they changed the paradigm of treatment regimen selection. In addition to the challenges of tumor heterogeneity at the time of initial diagnosis, acquired chemotherapy resistance further complicates our understanding and treatment of TNBC. Such genomic evolution of TNBC through chemotherapy selection pressure is well recognized but poorly understood. Large-scale genomic databases such as The Cancer Genome Atlas (TCGA) and Molecular Taxonomy of Breast Cancer International Consortium (METABRIC) have provided valuable resources for understanding the biology of primary BC tumors [12, 13]. However, there is a lack of data obtained from tumor specimens collected through longitudinal studies.

Analyzing the genomic and transcriptomic changes between paired primary and recurrent TNBC can provide useful insights to improve our understanding of the underlying tumor heterogeneity and tumor evolution with chemotherapy therapy selection pressure, and potentially lead to identification of novel therapeutic targets. The phosphoinositide-3-kinase (PI3K)/AKT/mammalian target of rapamycin (mTOR) pathway is one of the most commonly altered oncogenic pathways identified in BC. Activation of the PI3K/AKT/mTOR pathway contributes to resistance to chemotherapy [14–16]. Despite the high frequency of alterations of the PIK3CA/AKT/mTOR pathway, the presence of these mutations does not warrant a significant response to single agent PI3K or mTOR inhibitors in early clinical trials [17, 18].

Previous genomic analysis comparing primary and metastatic breast cancers have provided inconsistent findings [19]. Some indicated concordant overall mRNA expression patterns between primary in-breast tumors and matched lymph nodes [20–22], while others identified discordant mRNA expression patterns [23] or somatic mutation profiles [24] of in-breast tumor and synchronous metastases. The current study is designed to characterize the genomic and transcriptomic alterations in paired longitudinal samples of primary and recurrent TNBC, with a focus on the PI3K/AKT/mTOR pathway. Our finding of AKT3 upregulation provides a rationale for ATK targeting in treatment of metastatic TNBCs. To our knowledge this is one of the few studies analyzing both genomic and transcriptomic changes between longitudinal paired primary and metastatic TNBC.

## RESULTS

### Patient and disease characteristics (*N* = 10 patients)

**Figure 1:**
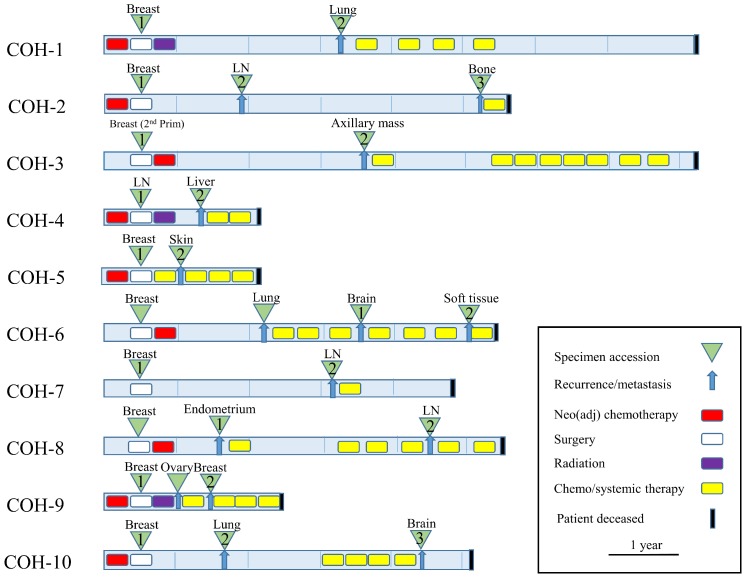
Clinical synopsis of treatment paradigm (*N* = 10 patients). TNBC specimens were collected at time of initial surgery and at first recurrence and/or metastasis (green triangles). Neo (adjuvant) chemotherapy, radiation, and lines of chemo/systemic therapy are also indicated.

The clinical characteristics, pathological features, treatment histories, and survival of a 10-pair TNBC cohort are described in Table 1. The majority of the tumors were infiltrating ductal carcinomas (IDC) (80%), stage I–III (90%), and the patients received standard-of-care chemotherapy with anthracycline and/or a taxane-containing regimen. Recurrence free survival (RFS) ranged from 2 to 39 months, and overall survival ranged from 9 to 92 months. Schematic paradigm of the treatment history and relapse pattern are shown in Figure 1, which illustrates the heterogeneity of treatment and duration of responses.

**Table 1:** TNBC patient characteristics, *N* = 10 patients

Patient ID	Tumor location	Histology type	Age	Stage	Neo(adjuvant) chemotherapy	Radiation	RFS month	OS month
COH-1.1	Breast primary	IDC	64	IIA	AC-T	Yes	34	89
COH-1.2	Lung met							
COH-2.1	Breast primary	IDC	51	IIIA	AC	Declined	17	60
COH-2.2	LN met							
COH-2.3	Bone met							
COH-3.1	Breast 2nd primary	IDC	41	IIIA	AC	Declined	39	92
COH-3.2	LN met							
COH-4.1	LN primary	IDC	44	IIIB	Carbo/Taxol	Yes	10	28
COH-4.2	Liver met							
COH-5.1	Breast primary	IDC	39	IIIC	AC	No	2	9
COH-5.2	Skin met							
COH-6.1	Brain met	IDC	50	IIA	TAC	Declined	31	67
COH-6.2	Soft tissue met							
COH-7.1	Breast primary	IDC	58	IA	Declined	Declined	26	46
COH-7.2	LN met							
COH-8.1	Endometrium met	IDC	38	IV	N.A.	No	11	58
COH-8.2	LN met							
COH-9.1	Breast primary	ILC	45	IIB	AC-T	Yes	8	23
COH-9.2	Breast met							
COH-10.1	Breast primary	Metaplastic	51	IIB	AC-T	Yes	16	58
COH-10.2	Lung met							
COH-10.3	Brain met							

LN, lymph node; RFS, relapse-free survival; OS, overall survival; IDC, Invasive ductal carcinoma; ILC, Invasive lobular carcinoma; AC, doxorubicin, cyclophosphamide; AC-T, doxorubicin, cyclophosphamide followed by docetaxel; Carbo/Taxol, carboplatin and paclitaxel.

### Genomic profiling of paired TNBCs (*N* = 7 patients)

**Figure 2:**
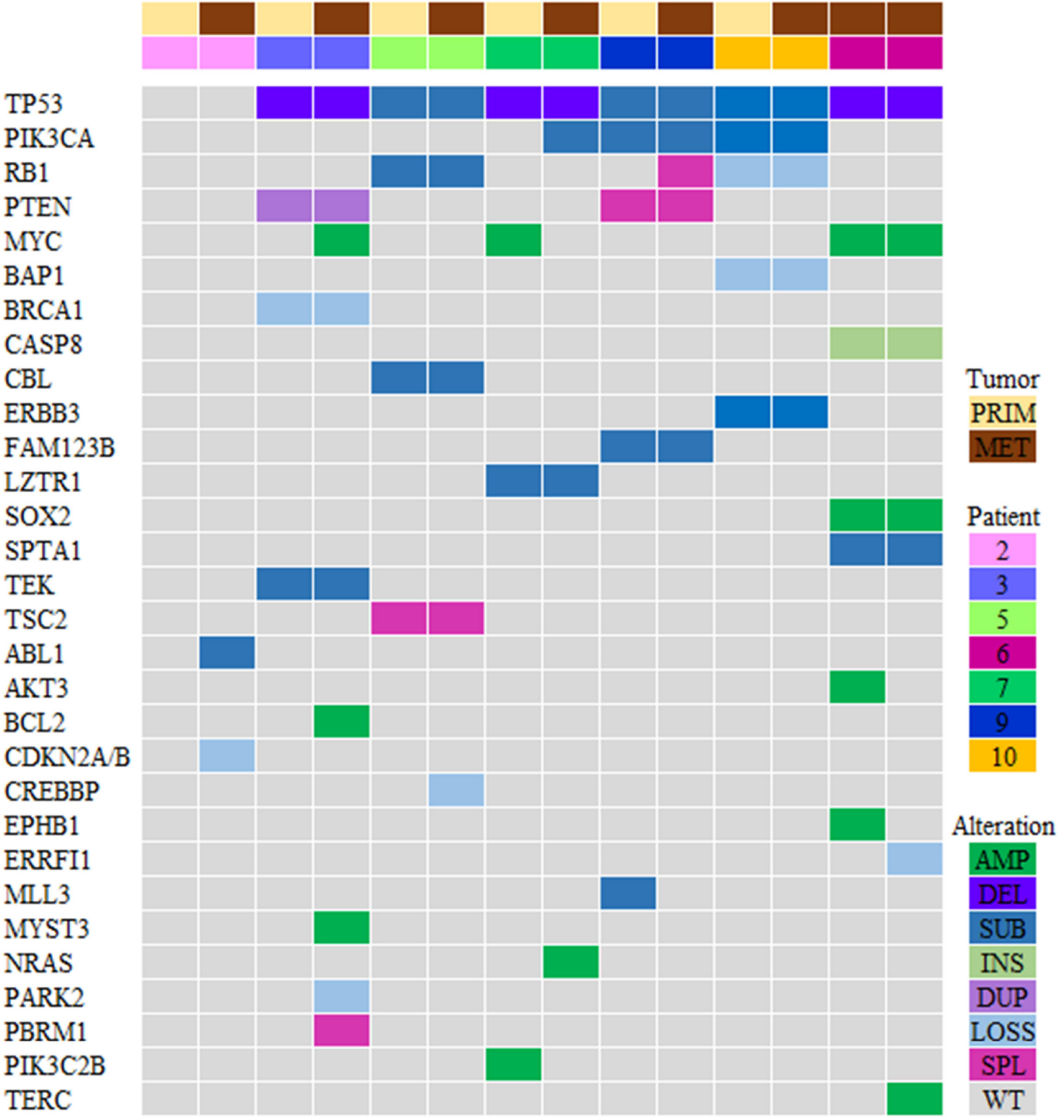
Genomic profiling of the PI3K pathway genes in TNBC (*N* = 7 patients). Tile plot illustrating patients ordered by primary/metastatic status (columns), and by mutation frequency (rows). Variants of unknown significance (VUS) were not included in this analysis. Prim, primary tumor; Met, metastatic tumor; Amp, amplification; Del, deletion; Sub, substitution; Ins, insertion; Dupl, duplication; Splice, splice site; WT, wild type.

Genomic sequencing was successful in 7 paired specimens (*N* = 14 specimens; Figure 2). Due to lack of sufficient tumor tissue, sequencing could not be performed for 3 patients. A total of 324 genomic alterations including 67 known mutations/amplifications and 257 variants of unknown significance (VUS) were identified. Genomic alterations were identified in the following signaling pathways: cell cycle, p53, PI3K/mTOR, RAS/MAPK, and RTK/GF [25]. The most abundant genomic alterations were found in the PI3K/AKT/mTOR pathway (PI3K/AKT genes were altered in 5 out of 7 patients in this cohort). There was significant inter-patient genomic heterogeneity, but little intra-patient variability. These findings not only confirm the genomic heterogeneity of TNBCs, but also highlight the genomic stability of these tumors over time.

### Transcriptomic profiling of paired TNBCs (*N* = 10 patients; 22 specimens)

**Figure 3:**
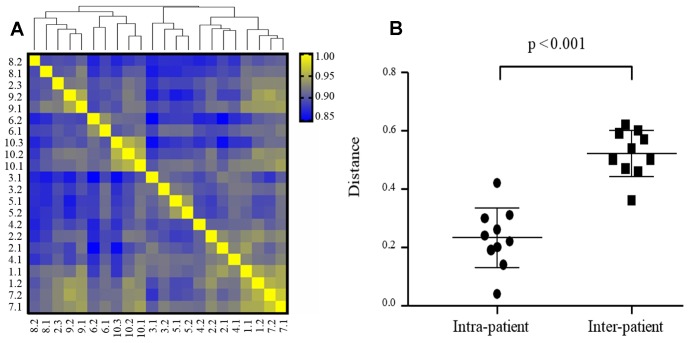
Intra- and inter-patient heterogeneity of mRNA expression in TNBC (*N* = 22 specimens). (**A**) Hierarchical cluster analysis of gene expression data (24,041 transcripts) from 22 TNBC specimens based on Pearson’s correlation. The color scale is based on the values of correlation coefficients. Distance between samples indicates the similarity of gene expression profiles. (**B**) mRNA expression is more concordant in intra-patient tumors than inter-patient tumors. Euclidian distances were calculated based on PC1, PC2, and PC3.

Principal coordinate analysis (PCoA) and hierarchical clustering were applied to assess the global expression pattern of the tumors, and showed clustering of samples for each patient. Only one distant metastasis COH-2.3 is dissimilar to COH-2.1 and COH-2.2, and more similar to COH-8 and COH-9 based on clustering (Figure 3A). The Euclidian distances analysis suggests that the mRNA expression is more concordant in intra-patient tumors than inter-patient tumors (Figure 3B).

### Hierarchical clustering of genes in the PI3 kinase (PI3K) pathway (*N* = 10 patients; 22 specimens)

**Figure 4:**
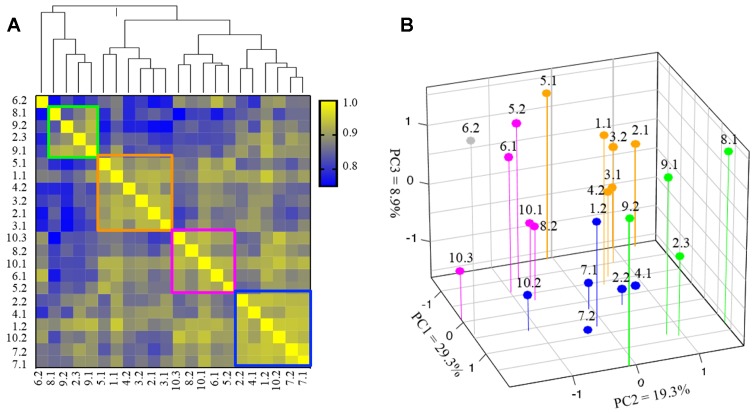
Subgroup clustering of PI3 kinase (PI3K) pathway genes (*N* = 22 specimens). (**A**) Hierarchical cluster analysis based on expression of 45 genes involved in PI3K pathway in 22 TNBC specimens. The color scale indicates the degree of correlation. Distance between samples indicate the similarity of gene expression profiles. Groups 1–4 are marked in green, orange, pink and blue boxes, respectively. COH-6.2 (gray) is an outlier and cannot be classified into any of the 4 groups. (**B**) PCoA based on gene expression involved in PI3K pathway (samples are color-coded into 4 groups as in 4A).

We next used hierarchical clustering to study the transcriptomic variation of PI3K pathway among the paired TNBCs. Four groups were identified in our clustering analysis, with one unique sample COH-6.2 (distant metastasis) that could not be classified into any other group. The samples classified in each group had higher values in Pearson correlation coefficients (Figure 4A). Specifically, the 22 samples were classified into Group 1 (green rectangle), consisting of 4 samples from 3 patients, Group 2 (orange rectangle) consisting of 6 samples from 5 patients, Groups 3 (pink rectangle) with 5 samples from 4 patients, and Group 4 (blue) with 6 samples from 5 patients. Noticeably, whereas the metastatic samples of patient COH-3, COH-7 and COH-9 were classified into the same groups as their primary cancer samples, the metastatic samples of 7 other patients (COH-1, COH-2, COH-4, COH-5, COH-6, and COH-10) were clustered into different groups. The samples of COH-2 were classified into Group 1, 2 and 4, and samples of COH-10 were classified into Group 3 and 4. PCoA analysis revealed similar classification of the 22 samples based on the expression of genes involved in the PI3K pathway (Figure 4B). The molecular subtyping using Lehmann/Pietenpol classifier was performed and there was no association between the subtypes and the 4 clusters. In addition, there was no association between the 4 clusters and disease/treatment variables or survival. Taken together, these results suggest that the gene expression profile of the PI3K pathway reveals four distinctive patterns in these heterogeneous TNBC specimens.

### Distinctive gene expression patterns in paired TNBCs

**Figure 5:**
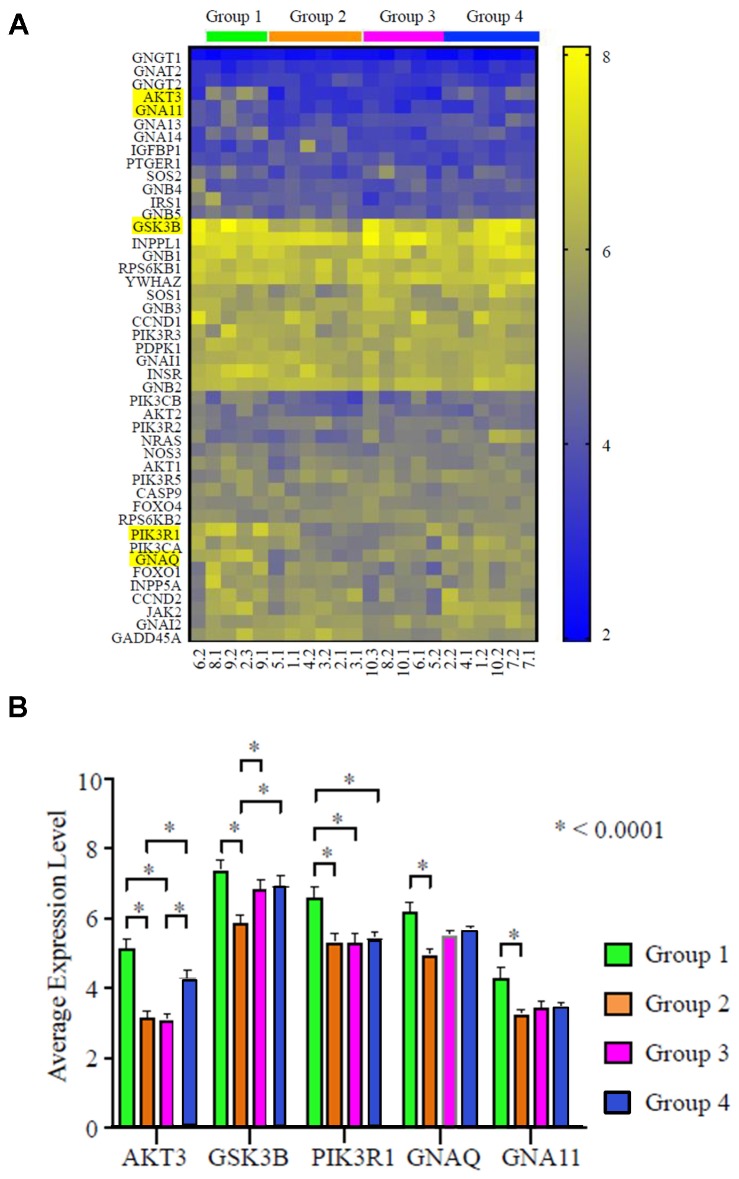
AKT3 is the most differentially expressed gene in the PI3K pathway in TNBC (*N* = 22 specimens). (**A**) Heat map of gene expression profile of the PI3K pathway. The four groups identified in Figure 3A are indicated with respective color bars labeled above the heat map. The top 5 genes with the largest expression variance are highlighted. (**B**) Relative mRNA expression level of the top 5 differentially expressed genes in the 4 respective groups; ^*^ *P* > 0.0001.

Further analysis was conducted to identify key genes within the PI3K/AKT/mTOR pathway which attributed to the distinctive expression pattern. Among all the differentially expressed genes, AKT3, GSK3B (glycogen synthase kinase-3 beta), PI3KR1 (phosphoinositide-3-kinase regulatory subunit 1), GNAQ (G protein subunit alpha Q), and GNA11 (G protein subunit alpha 11) showed the strongest statistical significance (*P*)>0.0001; Figure 5A and 5B). The expression level of AKT3 was highest in Group 1, followed by Group 4, Group 2, and lastly Group 3. The highest level of AKT3 expression is similar to the highest level of four other genes, including GSK3B, GNAQ, PIK3R1, and GNA11. These data suggest that AKT3, together with other genes in the PI3K signaling cascade, may be key players driving the molecular discordance in TNBC.

### Differentially expressed genes and survival in public datasets

The association of these 5 differentially expressed genes with patient’s survival were tested using public datasets. AKT1 and AKT2 were also studied. Using the mRNA expression dataset in Rody *et al.* (*N* = 64) [26], increased expression of AKT3 (*P* = 0.0896), GNA11 (*P* = 0.0369) and GNAQ (*P* = 0.0714) were associated with a trend of shorter disease free survival (Supplementary Figure 1). In METABRIC (*N* = 299), increased expression of AKT1 (*P* = 0.0312), GNA11 (*P* = 0.0639), and GSK3B (*P* = 0.0351) were associated with shorter overall survival (Supplementary Figure 2). There was no association of AKT1, AKT2, GNAQ and PIK3R1 with overall survival.

### AKT inhibitor ipatasertib is effective alone or in combination with chemotherapy in a PDX model of TNBC

**Figure 6:**
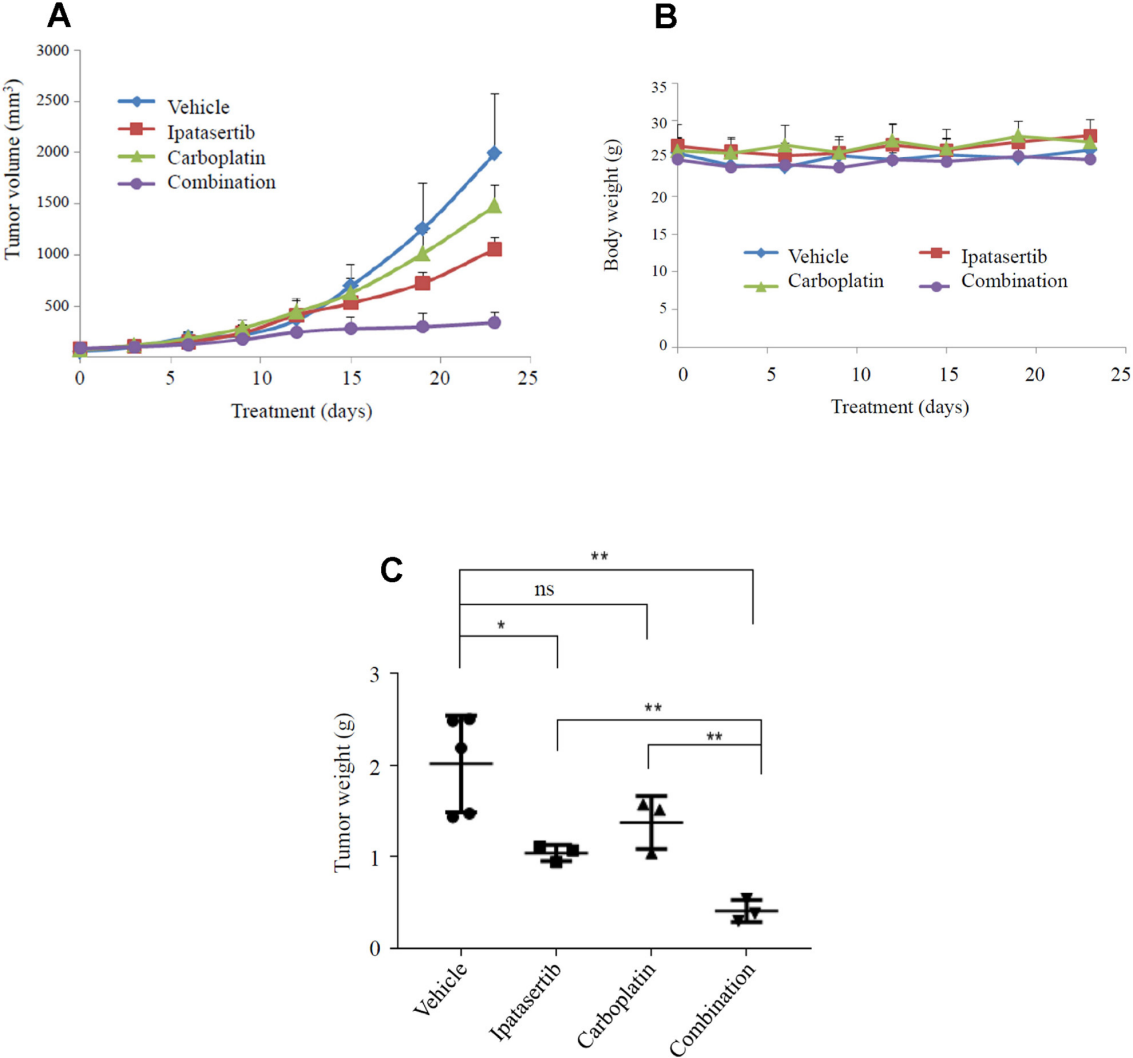
Synergistic effect of carboplatin and ipatasertib in a PDX model of TNBC. The dose of ipatasertib was 20 mg/kg for 9 days, followed by 30 mg/kg for 14 days; the dose of carboplatin was 10 mg/kg for two weeks, followed by 20 mg/kg for 1 week (total 3 doses). (**A**) PDX tumor volume changes over time; (**B**) Mice maintained their initial weight throughout the treatment; (**C**) Tumor volume among different groups: vehicle, ipatasertib, carboplatin, and combination ipatasertib plus carboplatin. ^*^ *P* = 0.05, ^**^ *P* = 0.005.

The synergistic effect of ipatasertib and carboplatin was tested *in vivo* using a TNBC patient-derived xenograft (PDX) with the following alterations: PIK3CA E542K mutation, PTEN loss and TP53 H179R mutation. Tumor volume changes over time within each treatment group are shown in Figure 6A. Of note, mice in all treatment groups maintained their initial weight over the course of treatment, as shown in Figure 6B. Statistically significant tumor suppression was seen in both ipatasertib alone (*P* = 0.05), and in the combination ipatasertib and carboplatin group (*P* = 0.005) (Figure 6C).

## DISCUSSION

Analysis of genomic and transcriptomic changes between paired primary and recurrent TNBCs may provide insight into the underlying tumor heterogeneity and tumor evolution with chemotherapy/radiation therapy selection pressure. A better understanding of these changes may ultimately lead to identification of potential targets for overcoming resistance. In this study, we identified 4 unique groups of TNBCs based on the PI3K/AKT/mTOR pathway genes. The AKT inhibitor ipatasertib was tested in a PDX model of TNBC with PI3K/PTEN alterations and synergistic tumor suppression confirmed the efficacy of AKT targeting. Our study demonstrates the importance of genomic analysis in assisting targeted therapy development.

The serine-threonine protein kinase AKTs are central proteins in many cellular pathways such as cell survival, proliferation, glucose uptake, metabolism, angiogenesis, as well as radiation and drug response [27]. The three isoforms of AKT (AKT1, AKT2 and AKT3) have been proposed to have different physiological functions, properties, and expression patterns depending on the cell types [28]. Knockout mouse studies have shown AKT1 to be essential for cell survival, AKT2 to have a more prevalent role in glucose homeostasis, while AKT3 is involved in brain development [29]. Little is known about the influence of the different AKT isoforms in the genome and their effects in TNBC.

The five genes identified to display distinctive expression patterns in this cohort have been shown to be closely implicated in cancer biology. AKT3 is involved in various biological processes such as proliferation, differentiation, apoptosis, and tumorigenesis [30]. Several studies have suggested AKT3 to be an oncogene and a potential therapeutic target in TNBC [1–3]. One study has further correlated the expression of AKT3 with metastasis of TNBC [3]. The AKT3 copy number gain is frequently observed in TNBC (26%, copy number > 4) and associated shorter RFS [31]. In line with these studies, we found that AKT3 is significantly overexpressed in groups 1 and 4 compared with groups 2 and 3 (Figure 5), suggesting that AKT3 may be an important driver in TNBC progression and chemotherapy-resistance. Furthermore, GSK3B, a serine-threonine protein kinase downstream of AKT3 showed a similarly distinctive expression pattern. Given the reported role of GSK3B in mitosis, proliferation, motility and survival [32], it is likely involved in AKT3-regulated TNBC biology. PIK3R1 phosphorylates inositol and indirectly activates AKT3. The activating mutations of PI3KCA, and PI3K signaling pathway-related genes have also been frequently observed, and are known to activate AKT3 [9]. GNAQ and GNA11 can form a Gq alpha subunit, and both are subunits of guanine nucleotide–binding proteins. Gq alpha interacts with cell membrane receptors and plays a role in signaling multiple pathways, especially growth signaling [4]. Activating Q209L/P mutations in *GNAQ or GNA11* (GNAQ/11) are present in approximately 5.6% of tumors and 80% of uveal melanomas. Combination of small molecules inhibiting MEK and PI3K enhances uveal melanoma cell death in a mutant *GNAQ/GNA11*-dependent manner [33, 34]. A phase II study with selumetinib (an ATP-independent inhibitor of mitogen-activated protein kinase) showed improved clinical activity compared with temozolomide in GNAQ/GNA11 mutant uveal melanoma [35]. Taken together, our findings suggest that the discordant expression of these genes may underlie the heterogeneity of TNBC and can serve as targets for novel combinations.

AKT targeting has made progress in recent clinical trials. Pan-AKT inhibitors have shown promising responses in patients with metastatic TNBC [36–38]. Ipatasertib is a highly selective ATP-competitive small-molecule AKT inhibitor, and showed activity in cell lines and xenograft models of a broad range of cancer types including breast cancer [39]. Sensitivity to ipatasertib was associated with high phosphorylated AKT levels, PTEN protein loss, or genetic mutations in PTEN and PIK3CA, whereas KRAS and BRAF mutations were typically associated with resistance to ipatasertib [39]. In a recent update of the Phase II LOTUS trial, paclitaxel in combination with ipatasertib showed a higher response rate and more durable responses in patients with TNBC tumors harboring PI3K/AKT/PTEN alterations [39].

DNA-damaging agents such as platinum drugs (cisplatin and carboplatin) are active in TNBC. In the TNT trial, patients with TNBC and germline BRCA1 and/or BRCA2 mutations were found to have a higher response rate and longer progression-free survival rate favoring carboplatin over docetaxel [40]. Our data from this PDX model showed promising synergistic effects combining ipatasertib and carboplatin. Hence, there is a strong rationale for testing this combination in the clinical setting. A phase II clinical trial combining ipatasertib and carboplatin is currently ongoing.

Our study is limited by its small sample size due to the challenge of obtaining longitudinal specimens over a long time course. Future studies elucidating the impact and biological significance of the 4 distinctive patterns of PI3K/AKT/mTOR pathway seen in this study are currently in progress.

Differential gene expression between paired primary and metastatic TNBCs was observed in this 10 patient cohort, with an increase in growth-promoting signals. Among the genes involved in PI3K pathway alterations, AKT3 appears to play a critical role and is a potential target for novel therapies for metastatic TNBC. Our mTNBC PDX model further confirms AKT as a potential target for treatment of TNBC. A phase I/II clinical is planned for further assessment of the clinical activity of this combination.

## MATERIALS AND METHODS

### Patient selection

Longitudinal paired primary and metastatic TNBC specimens were identified through an Institutional Review Board (IRB)-approved protocol from patients with recurrence between 2002 and 2018. The eligibility criteria were: stage I–III breast cancer; ER negative, PR negative and HER-2neu negative defined by ASCO/CAP guideline; at least one tumor biospecimen available from initial surgery and metastatic biopsy. A total of 10 patients were studied in the current cohort. Of these, 2 patients had 3 samples each, resulting in a total of 22 specimens in the study. All pathology samples were formalin-fixed and paraffin-embedded (FFPE). Demographic data such as age, gender, date of birth, date of diagnosis, date of relapse, and date of death or last follow-up (if applicable) were obtained. Disease characteristics such as tumor grade, TNM stage, and ER/PR/HER2 status, as well as treatment variables including surgery, chemotherapy, and radiation therapy were also obtained.

### mRNA expression of primary and recurrent TNBCs

Messenger RNA (mRNA) expression was profiled using GeneChip^®^ Human Gene 2.0 ST array. Raw data were normalized and processed using Expression Console, and linear regression was performed using Limma to identify the differentially expressed genes between primary and recurrent/refractory TNBC. The expression of 24,041 transcripts was normalized by robust multi-array average (RMA) using R Bioconductor “oligo” package [41–44]. The annotation of the genes was constructed based on R Bioconductor “AnnotationDbi” package [45]. Gene expression was summarized by max approach. The similarity among samples was calculated based on Pearson correlation coefficients, which were used to construct hierarchical clustering and heat maps. Pathway-related heat maps were constructed, and hierarchical clustering based on the gene expression correlation of the genes defined in the pathways of Panther database (V3.4.1) were analyzed according to the methods described [46]. Pathways with differential gene conserved expression pattern were selected. The genes of the PI3 kinase pathway (P00048) defined in the Panther database (V3.4.1) [46] were selected for further analysis. The Principal Component Analysis (PCA) and Principal Coordinates Analysis (PCoA) were performed using the methods described in Past 3.14 [47]. The Euclidian distances between samples were calculated based on the coordinates of PC1, PC2 and PC3. The P-value of differential gene expression between the different groups of PI3K pathways were calculated by 2-way ANOVA multiple comparison using GraphPad Prism 7.

### Gene expression and survival analyses using public databases

Gene expression and survival data of 64 patients with TNBC from Rody, *et al*. [26] and 299 patients with TNBC from METABRIC [12] were downloaded. The “high” and “low” groups were separated based on median mRNA expression values. Kaplan–Meier survival analyses, and were used to determine the survival differences between “high” and “low” mRNA expression groups. *P*-values were calculated by log-rank test using the Survival package in R [48]. The survival differences were considered to be statistically significant when *P*-values were > 0.05.

### Akt inhibitor ipatasertib and carboplatin in patient derived xenograft (PDX) model

After obtaining informed written patient consent, TNBC tumor samples were obtained from patients at the time of surgery or biopsy at COH under protocol approved by COH Institutional Review Board (IRB). Fresh primary tumor tissues (2–3 mm in diameter) were surgically implanted into the mammary fat pad of 6- to 8-week-old female NOD/SCID/IL2Rgamma-null (NSG) mice. Once the xenograft was established, the tumor was removed, cut into small fragments, and subsequently passaged from mouse to mouse to expand the xenograft number. These mice were then used for the experiment. When the xenografts were palpable, animals were randomized into 4 groups and treated daily by oral gavage with vehicle, carboplatin, ipatasertib or a combination of both agents. Tumor volumes were assessed using calipers one to two times a week. The dose of ipatasertib was 20 mg/kg for 9 days, followed by 30 mg/kg for 14 days; the dose of carboplatin was 10 mg/kg for two weeks, followed by 20 mg/kg for 1 week (total 3 doses). The mice were sacrificed at day 23. Tumor volumes were calculated using the formula (width) ^2^ × length × 0.52. Body weight was monitored weekly as an indicator of drug-induced toxicity and overall health of the mice. All animal studies were carried out under protocols approved by the Institutional Animal Care and Use Committee (IACUC) at COH in accordance with all applicable federal, state, and local requirements and institutional guidelines.

### Ethics

All procedures performed in studies involving human participants were in accordance with the ethical standards of the institutional and/or national research committee, and with the 1964 Helsinki declaration and its later amendments or comparable ethical standards. Informed consent was obtained from all participants included in the study. All tumor specimens were identified through a City of Hope IRB-approved retrospective protocol from patients consented to City of Hope Biorepository Protocol.

## SUPPLEMENTARY MATERIALS



## Abbreviations

AC: Adriamycin/cyclophosphamide
AC-T: Adriamycin/cyclophosphamide followed by paclitaxel
Amp: Amplification
BC: Breast cancer
Carbo/paclitaxel: Carboplatin/paclitaxel
Del: Deletion
Dupl: Duplication
FFPE: Formalin-fixed and paraffin-embedded
IDC: Infiltrating ductal carcinomas
Ins: Insertion
IRB: Institutional Review Board
ILC: Invasive lobular carcinoma
LN: Lymph node
mTOR: Mammalian target of rapamycin
mRNA: Messenger RNA
Met: Metastatic tumor
METABRIC: Molecular Taxonomy of Breast Cancer International Consortium
NSG: NOD/SCID/IL2Rgamma-null
OS: Overall survival
PDX: Patient-derived xenograft
PI3K: Phosphoinositide-3-kinase
Prim: Primary tumor
PCA: Principal Component Analysis
PCoA: Principle coordinate analysis
PD-L1: Programmed death ligand-1
RFS: Relapse-free survival
RMA: Robust multi-array average
Splice: Splice site
Sub: Substitution
TCGA: The Cancer Genome Atlas
TNBC: Triple negative breast cancer
VUS: Variants of unknown significance
WT: Wild type
