# Supplementary material for: Differential gene expression and AKT targeting in triple negative breast cancer
Source: Oncotarget. 2019 Jul 9;10(43):4356–68. doi: 10.18632/oncotarget.27026 (PMC6633890; doi:10.18632/oncotarget.27026)
Supplement: Supplementary file 1 [file oncotarget-10-4356-s001.pdf]

Differential gene expression and AKT targeting in triple negative breast cancer

SUPPLEMENTARY MATERIALS

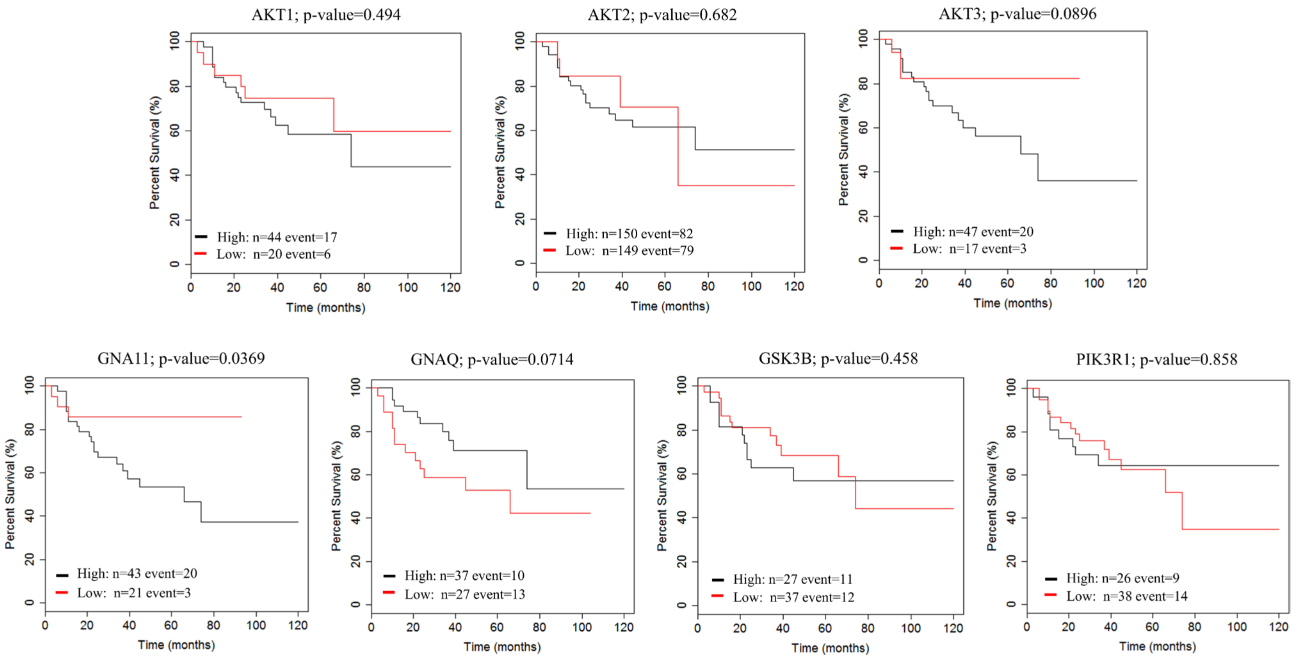

Supplementary Figure 1: Gene expression level and overall survival in TNBCs from Rody *et al.* (*N* = 64). Higher expression of AKT3 (*P* = 0.0896) GNA11 (*P* = 0.0369) and GNAQ (*P* = 0.0714) are associated with trends of shorter disease free survival.

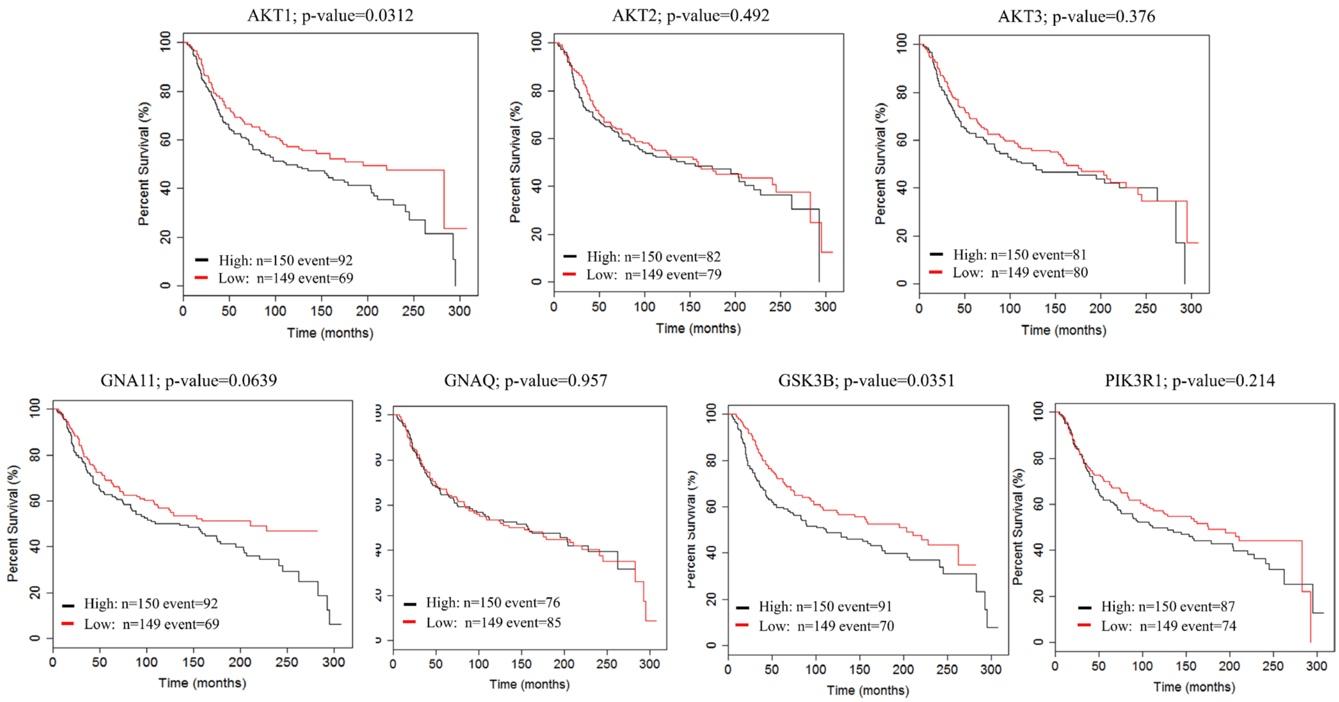

Supplementary Figure 2: Gene expression level and overall survival in METABRIC TNBCs (*N* = 299). Higher expression of AKT1 (*P* = 0.0312), GNA11 (*P* = 0.0639), and GSK3B (*P* = 0.0351) are associated with trends of shorter overall survival.
